# Prediction of carotid vulnerable plaques in patients with type 2 diabetes using interpretable machine learning models

**DOI:** 10.3389/fendo.2026.1911126

**Published:** 2026-08-18

**Authors:** Qi Cheng, Fengjuan Zhang, Han Wang, Yi Qian, Ping Tang, Weiming Cai, Pingbo Chen, Dengke Li, Lijie Shi

**Affiliations:** 1 Jingjiang People's Hospital Affiliated to Yangzhou University, Taizhou, China; 2 Yijishan Hospital of Wannan Medical College, Wuhu, China; 3 Suzhou Hospital of Anhui Medical University, Suzhou, China

**Keywords:** carotid vulnerable plaque, continuous glucose monitoring, machine learning, shapley additive explanations, type 2 diabetes mellitus

## Abstract

**Objective:**

To develop and externally validate an interpretable machine-learning framework for estimating ultrasound-defined carotid plaque vulnerability among patients with type 2 diabetes mellitus (T2DM) and established carotid plaque.

**Methods:**

In total, 884 T2DM patients with carotid atherosclerotic plaques from two medical centers were retrospectively recruited and allocated into training, internal validation, and external validation cohorts. Demographic, clinical, biochemical, continuous glucose monitoring (CGM), and inflammatory indicators were collected. CGM was performed for 72 consecutive hours using a retrospective CGM system (Medtronic iPro2; Medtronic, Northridge, CA, USA), and TIR was calculated from valid CGM recordings. Data preprocessing included clinically reviewed missing-data handling, standardization of continuous variables, and Synthetic Minority Over-sampling Technique (SMOTE), which was fitted only within the training data and, during cross-validation, within each training fold to avoid information leakage. Core predictors were identified by integrating feature-importance rankings derived from Random Forest, linear Support Vector Machine, and Logistic Regression. Five predictive models were developed and evaluated using discrimination, calibration, and clinical-utility metrics.

**Results:**

Six core predictors were identified: systolic blood pressure (SBP), low-density lipoprotein cholesterol (LDL-C), age, time in range (TIR), systemic immune-inflammation index (SII), and smoking status. The XGBoost model showed the best overall validation performance, attaining an AUC of 0.882 (95% CI: 0.795-0.969) in the external validation set. SHAP analysis indicated that higher SBP, higher LDL-C, older age, higher SII, and smoking were associated with a higher predicted probability of vulnerable plaques, whereas higher TIR was associated with a lower predicted probability.

**Conclusion:**

An interpretable XGBoost model based on six clinical, laboratory, and CGM-derived indicators showed acceptable discrimination, calibration, and clinical net benefit for estimating ultrasound-defined plaque vulnerability among patients with T2DM and established carotid plaque. The model is not a substitute for carotid ultrasound; pending prospective workflow and economic evaluation, it may support research-stage prioritization for expert plaque characterization when imaging capacity or expertise is constrained.

## Introduction

Carotid atherosclerosis represents an important pathological substrate of ischemic stroke. The clinical risk associated with this condition is dictated not only by the degree of luminal stenosis but also by the biological stability of atherosclerotic plaques. Vulnerable plaques are typically distinguished by intraplaque hemorrhage, extensive lipid necrotic cores, thin or ruptured fibrous caps, surface ulceration, and neovascularization (1–3). Type 2 diabetes markedly accelerates atherosclerosis and elevates cardiovascular risk (4, 5). Long-term hyperglycemia impairs vascular endothelial function, aggravates systemic inflammatory responses, and disturbs lipid metabolism, leading to free fatty acid and triglyceride accumulation. These factors may promote plaque progression and adverse vascular events (6, 7). Rupture of vulnerable plaques followed by thrombus formation or embolization can cause cerebral ischemia (8). Therefore, identifying carotid plaque vulnerability in patients with T2DM is clinically relevant for imaging prioritization and vascular risk assessment, while its effect on future stroke prevention requires prospective outcome evaluation.

Carotid ultrasound is the first-line non-invasive method for visualizing plaque morphology, but vulnerability assessment remains dependent on image acquisition, reader expertise, and standardized interpretation; reproducible grading is not always achieved even among experienced observers (9). CTA and MRI can provide additional characterization but are less suitable for broad first-line use because of radiation or contrast exposure, cost, and limited availability. The clinical problem is therefore not that ultrasound lacks diagnostic value, but that universal expert-level plaque characterization is not always feasible. Nationally representative data on the proportion of Chinese patients with T2DM who undergo carotid ultrasound are not currently available. Current Chinese primary-care guidance specifies routine assessment of established diabetic complications and peripheral vascular disease but does not establish universal carotid ultrasonography for all asymptomatic patients with T2DM (10). Consistently, population-wide screening for asymptomatic carotid stenosis is not recommended by the US Preventive Services Task Force because a net clinical benefit has not been demonstrated (11). These considerations support a targeted-imaging strategy rather than indiscriminate ultrasound screening.

Within such a targeted strategy, a non-imaging model could add value by estimating the likelihood of plaque vulnerability from systemic risk information before dedicated expert plaque characterization. This does not replace carotid ultrasound or establish a diagnosis. Rather, it could help prioritize limited expert-ultrasound or advanced-imaging capacity among patients with T2DM who already have carotid plaque documented and require vulnerability characterization. A second potential use is as an adjunct when a basic ultrasound report is equivocal or produced in a setting with limited plaque-characterization expertise; this use remains hypothetical. Because the present cohort required ultrasound-confirmed carotid plaque for inclusion, the model has not been validated as a universal screening tool for all previously unscreened patients with T2DM.

In recent years, machine learning (ML) has exhibited strong advantages in processing high-dimensional data, modeling nonlinear relationships, and constructing high-precision predictive models, leading to its widespread application in cardiovascular and endocrine diseases (12–14). Previous investigations have verified the effectiveness of ML in detecting carotid plaques and assessing their vulnerability (15–18). Nevertheless, most existing studies have largely overlooked the high-risk T2DM population and failed to incorporate continuous glucose monitoring (CGM) metrics alongside systemic inflammation indices. These omissions restrict the specificity and clinical applicability of the resulting models.

In this investigation, we combined CGM, clinical, biochemical, and inflammatory parameters to construct an ML model for estimating the probability of ultrasound-defined carotid vulnerable plaque among patients with T2DM and established carotid plaque. Ultrasound served as the reference standard, and the model underwent internal validation and independent external validation, with interpretability assessed using the SHAP method. The study was designed to test non-imaging risk enrichment before expert vulnerability characterization, not to replace ultrasound, diagnose carotid disease in all patients with T2DM, establish causal relationships, or predict future stroke events.

## Methods

### Study population

**Figure 1:**
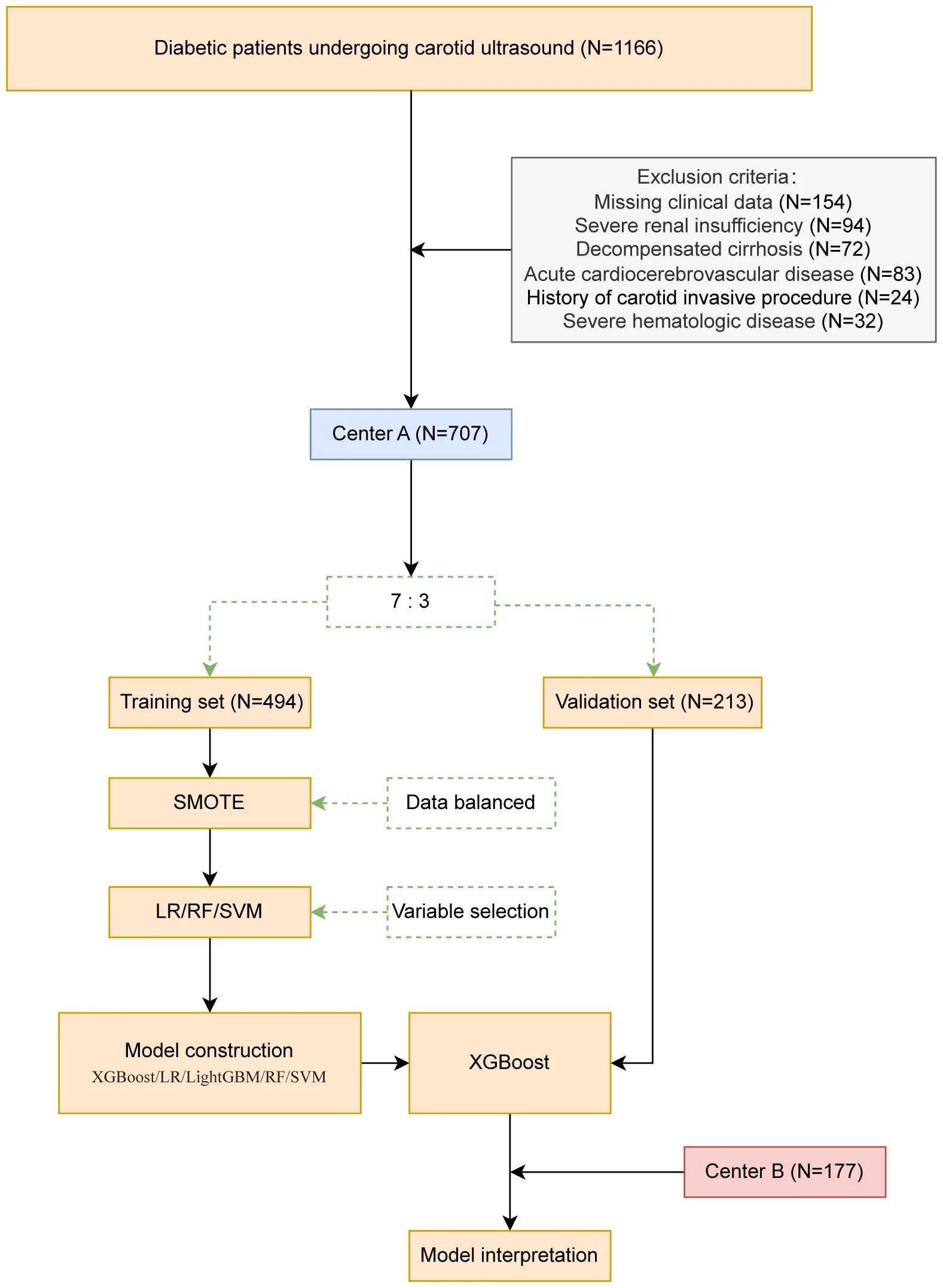
Flowchart of patient selection and study design.

This retrospective observational investigation consecutively recruited 707 patients with T2DM and carotid atherosclerotic plaques from Yijishan Hospital of Wannan Medical University between August 2020 and August 2024. Participants were randomly assigned to a training set (n=494) and an internal validation set (n=213) at a 7:3 ratio. Furthermore, 177 T2DM patients with carotid plaques from Suzhou Hospital of Anhui Medical University were recruited as an external validation set to evaluate model generalizability. Comprehensive inclusion and exclusion criteria are presented in **Figure 1**. Patients were excluded before model construction when key eligibility or outcome information was unavailable, including absent plaque classification, unavailable CGM records, or missing essential clinical information that precluded eligibility assessment. Sporadic missingness in retained candidate predictors was subsequently handled by multiple imputation, as described below. The study was performed in accordance with the Declaration of Helsinki and the TRIPOD reporting principles for clinical prediction models. Ethical approval was secured from the Ethics Committees of Yijishan Hospital of Wannan Medical University and Suzhou Hospital of Anhui Medical University. As this was a retrospective study involving no additional interventions, the Ethics Committee waived the informed consent requirement.

**Figure 2:**
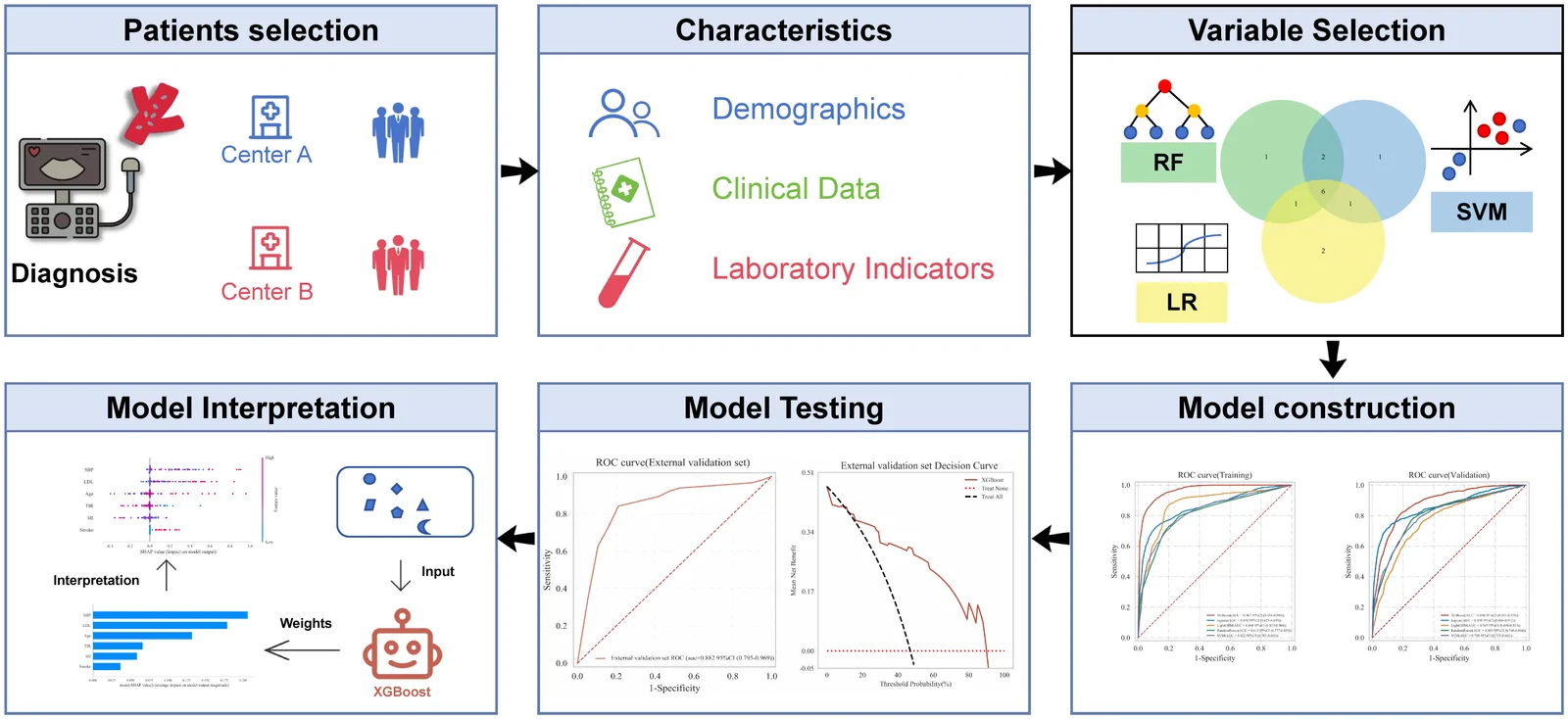
Flowchart of the data processing and modeling strategy.

All participants received a carotid ultrasound examination to verify atherosclerotic plaque presence. Vulnerable plaques were defined as plaques demonstrating hypoechoic characteristics, with or without an irregular plaque surface contour, an incomplete fibrous cap, or an intra-plaque blood-flow signal suggestive of ulceration. Stable plaques were defined as plaques with isoechoic or hyperechoic characteristics, a regular plaque surface, and an intact fibrous cap (19, 20). All plaque assessments were conducted independently and blindly by two physicians with more than 5 years of cardiovascular ultrasound expertise. Inter-observer agreement before adjudication was assessed using Cohen’s kappa. In instances of inconsistency, a third senior physician reviewed the images and determined the final classification. In this manuscript, “expert plaque-focused ultrasound” refers to a dedicated conventional carotid duplex assessment in which experienced vascular sonographers and readers use protocolized high-resolution B-mode and color-Doppler imaging to characterize plaque echogenicity, surface regularity or ulceration, fibrous-cap integrity, and intra-plaque flow. This differs in purpose from a standard carotid duplex examination, which primarily assesses arterial patency, flow velocities, and stenosis and may provide only a limited plaque description. The plaque-focused examination may require additional acquisition and interpretation time and specialized expertise. It does not denote contrast-enhanced ultrasound (CEUS), and no microbubble contrast agent was used in this study. The study workflow is depicted in **Figure 2**.

### Data collection

The collected variables included demographic data, clinical indicators, laboratory biochemical parameters, CGM metrics, and inflammation-related indicators. Potential predictors were initially determined according to the study design and clinical expert opinions. After data preprocessing and the removal of features with a missing-value ratio above 20%, the following variables were retained for analysis: age, sex, body mass index (BMI), drinking history, smoking history, diastolic blood pressure (DBP), systolic blood pressure (SBP), fasting plasma glucose (FPG), C-peptide (CP), glycated hemoglobin A1c (HbA1c), triglycerides (TG), total cholesterol (TC), low-density lipoprotein cholesterol (LDL-C), high-density lipoprotein cholesterol (HDL-C), uric acid (UA), serum creatinine (SCr), estimated glomerular filtration rate (eGFR), urine albumin-to-creatinine ratio (UACR), glucose management indicator (GMI), time in range (TIR), mean blood glucose (MBG), time above range (TAR), time below range (TBR), coefficient of variation (CV), standard deviation of blood glucose (SD), mean amplitude of glycemic excursions (MAGE), lymphocyte count (Lc), neutrophil count (Nc), platelet count (PLT), systemic immune-inflammation index (SII), and neutrophil-lymphocyte ratio (NLR). All laboratory indicators were measured using an automated biochemical analyzer (Cobas 8000 c701, Roche Diagnostics, Mannheim, Germany). Homocysteine was available in 339 of 884 patients (38.3%) and was missing in 545 patients (61.7%); therefore, it was not included because it was not routinely measured across both centers and had substantial missingness in the available records.

CGM protocol. CGM was performed using a retrospective CGM system (Medtronic iPro2; Medtronic, Northridge, CA, USA) for 72 consecutive hours. For all participants included in this retrospective study, the CGM assessment had been obtained for routine clinical diabetes-management assessment during hospitalization and was already available in the clinical record; CGM was not initiated for this research. Thus, among the included patients, 100% of CGM assessments were clinically obtained and 0% were research-specific. Only valid CGM recordings with adequate wear time were used for metric calculation. TIR was defined as the percentage of time spent within 3.9-10.0 mmol/L, TAR as the percentage of time above 10.0 mmol/L, and TBR as the percentage of time below 3.9 mmol/L. GMI, MBG, SD, CV, and MAGE were calculated from valid CGM data according to standard CGM metrics.

### Data preprocessing

Before imputation, all variables were checked for completeness and plausibility. Variables with a missing-value ratio above 20% were excluded from candidate predictors. For retained variables, sporadic missing values were addressed through multiple imputation with five imputations to construct complete datasets for statistical inference and model development (21, 22). Continuous variables were screened using both statistical and clinical plausibility criteria. Values beyond the 1.5 interquartile-range limits were flagged for review rather than automatically removed. Flagged values were checked against source records and clinically plausible extremes were retained. Only physiologically impossible values or confirmed data-entry errors that could not be recovered from source records were treated as missing values and included in the imputation process. Multiple imputation was performed separately within the training, internal validation, and external validation cohorts to avoid information leakage. Missingness percentages for retained predictors are summarized in **Supplementary Table 2**.

The ratio of stable plaques to vulnerable plaques in this study was approximately 4:1, indicating class imbalance. To improve recognition of the minority class (vulnerable plaques), SMOTE was applied only to the training data. During 5-fold cross-validation and grid-search tuning, SMOTE was embedded in the modeling pipeline and fitted independently within each training fold after the cross-validation split; the held-out fold was not oversampled. The internal validation and external validation cohorts were never oversampled. This pipeline-based implementation was used to reduce data leakage and synthetic-sample bias. To further evaluate the influence of SMOTE, sensitivity analyses comparing model performance before and after SMOTE were added in **Supplementary Table 3** (23).

### Feature selection

To identify robust predictive factors, a multi-model fusion strategy for feature-importance evaluation was adopted. Three algorithms-Random Forest (RF), linear Support Vector Machine (SVM), and Logistic Regression (LR)-were used to compute and rank each variable’s predictive contribution. RF captured nonlinear and interaction-based contributions through Gini-index reduction, whereas linear SVM and LR captured linear margin- and coefficient-based associations. After extracting the top 10 important features from each algorithm, the common features identified by all three models were chosen as final core predictors. This intersection-based strategy reduces dependence on a single algorithm, improves feature-selection stability, limits model complexity relative to the number of vulnerable-plaque cases, and enhances clinical interpretability.

### Model development and evaluation

Five ML models were developed using the training dataset: XGBoost, LightGBM, RF, SVM, and LR. Grid search with 5-fold cross-validation was used for hyperparameter tuning. All preprocessing steps that required data fitting, including standardization and SMOTE, were fitted only within the training portion of each cross-validation fold and then applied to the held-out fold. Model discrimination was primarily assessed using the area under the receiver operating characteristic curve (AUC). Moreover, accuracy, sensitivity, specificity, positive predictive value (PPV), negative predictive value (NPV), and F1-score were computed to comprehensively examine model classification performance. For calibration, calibration curves were created to visually demonstrate concordance between predicted probabilities and observed probabilities, and the Brier score was used for quantitative assessment. Decision Curve Analysis (DCA) was performed to evaluate clinical net benefit across threshold probabilities. Bootstrap resampling was used to estimate 95% confidence intervals for key performance metrics, and optimism-corrected estimates were added as sensitivity analyses in **Supplementary Table 4** (24).

### External validation

An independent external cohort from a different medical center was used to evaluate model transportability. Because the external cohort contained a limited number of vulnerable-plaque cases, external validation results were interpreted with attention to confidence intervals and calibration uncertainty.

### Model interpretability

SHAP (SHapley Additive exPlanations) values were employed to assess each feature’s marginal contribution to prediction outcomes, providing both global and individual-level model interpretation (25).

### Statistical analysis

Statistical analyses were performed using SPSS version 26.0 and Python. The normality of continuous variables was evaluated using the Shapiro-Wilk test. Normally distributed continuous variables were expressed as mean +/- standard deviation (SD), with between-group comparisons performed using independent-samples t-tests. Non-normally distributed continuous variables were reported as median (interquartile range, IQR), with between-group comparisons performed using the Mann-Whitney U test. Categorical variables were displayed as frequencies (percentages, %), with between-group comparisons conducted using the chi-square test or Fisher’s exact test. Age was retained as a continuous variable in all feature-selection and model-development procedures and was not dichotomized or categorized. When scaling was required by an algorithm, the transformation was fitted only within the training portion of each cross-validation fold. Inter-observer agreement for ultrasound-defined plaque vulnerability was quantified using Cohen’s kappa before adjudication. Bootstrap resampling was used to calculate confidence intervals for model-performance metrics where applicable. The software and packages used for data preprocessing, model construction, validation, and interpretability analysis are shown in **Supplementary Table 1**.

## Results

### Baseline characteristics

In total, 707 T2DM patients with carotid atherosclerotic plaques were recruited from Yijishan Hospital of Wannan Medical University. Among them, 134 patients (19.0%) presented with vulnerable plaques, while 573 patients (81.0%) presented with stable plaques. The training set, internal validation set, and external validation set’s demographic and clinical baseline characteristics are detailed in **Table 1**.

**Table 1:** Baseline characteristics of study participants.

Characteristics	Training set (n=494)			Internal validation set (n=213)			External validation set (n=177)		
	Stable plaque (n=403)	Vulnerable plaque (n=91)	p	Stable plaque (n=170)	Vulnerable plaque (n=43)	p	Stable plaque (n=148)	Vulnerable plaque (n=29)	p
Gender, n (%)			0.990			0.497			0.073
Female	230 (57.072)	52 (57.143)		93 (54.706)	26 (60.465)		88 (59.459)	12 (41.379)	
Male	173 (42.928)	39 (42.857)		77 (45.294)	17 (39.535)		60 (40.541)	17 (58.621)	
Smoke, n (%)			<0.001			0.040			0.002
No	349 (86.600)	61 (67.033)		139 (81.765)	29 (67.442)		120 (81.081)	16 (55.172)	
Yes	54 (13.400)	30 (32.967)		31 (18.235)	14 (32.558)		28 (18.919)	13 (44.828)	
Drink, n (%)			0.693			0.239			0.146
No	309 (76.675)	68 (74.725)		126 (74.118)	28 (65.116)		124 (83.784)	21 (72.414)	
Yes	94 (23.325)	23 (25.275)		44 (25.882)	15 (34.884)		24 (16.216)	8 (27.586)	
Age (years)	62.700 [57.000,66.000]	72.600 [66.000,77.000]	<0.001	62.700 [57.000,67.000]	71.000 [65.000,82.000]	<0.001	62.000 [56.000,65.000]	76.000 [71.000,79.000]	<0.001
BMI (kg/m^2^)	26.028 [24.112,28.395]	25.510 [23.110,27.606]	0.050	26.030 [24.000,27.487]	26.037 [24.088,27.540]	0.917	26.028 [24.559,27.729]	24.490 [23.300,26.733]	0.069
SBP (mmHg)	131.000 [120.000,137.300]	144.000 [132.000,158.000]	<0.001	129.500 [118.000,136.100]	147.000 [134.000,157.000]	<0.001	130.000 [119.000,136.000]	141.900 [129.000,147.000]	<0.001
DBP (mmHg)	85.100 [79.900,93.000]	85.000 [75.000,91.600]	0.091	85.000 [79.000,91.000]	85.000 [79.000,91.000]	0.739	86.149 ± 8.398	82.514 ± 8.964	0.038
FPG (mmol/L)	8.280 [6.900,9.600]	8.340 [7.030,10.120]	0.633	8.380 [7.400,9.740]	8.704 [7.600,9.764]	0.430	8.791 [7.460,10.140]	8.947 [6.980,10.796]	0.929
CP (μg/L)	1.540 [1.100,2.000]	1.600 [0.900,2.170]	0.826	1.460 [1.000,1.900]	1.540 [0.900,1.900]	0.857	1.500 [1.100,1.900]	1.600 [1.100,1.900]	0.953
HbA1c (%)	8.590 [7.000,9.700]	8.400 [7.300,9.700]	0.675	8.600 [7.200,9.500]	9.000 [8.300,10.600]	0.021	8.800 [7.700,9.700]	8.600 [7.300,10.000]	0.768
TC (mmol/L)	4.820 [4.185,5.370]	4.670 [3.850,5.590]	0.374	4.900 [4.350,5.310]	4.560 [4.030,5.040]	0.034	4.770 [4.251,5.280]	4.400 [3.520,4.894]	0.053
TG (mmol/L)	2.020 [1.390,3.029]	2.140 [1.650,3.100]	0.439	2.110 [1.450,3.316]	2.240 [1.600,3.010]	0.482	2.410 [1.760,3.815]	2.130 [1.550,2.497]	0.216
HDL-C (mmol/L)	1.200 [0.970,1.420]	1.260 [1.060,1.570]	0.045	1.200 [0.970,1.390]	1.219 [1.000,1.413]	0.852	1.189 ± 0.279	1.301 ± 0.352	0.063
LDL-C (mmol/L)	2.760 [2.320,3.226]	3.220 [2.460,3.810]	<0.001	2.791 [2.330,3.260]	3.180 [2.520,3.714]	0.021	2.748 ± 0.707	3.046 ± 0.812	0.046
UA (μmol/L)	294.700 [253.100,343.000]	288.000 [234.000,347.000]	0.217	282.900 [242.000,336.000]	288.000 [235.000,318.300]	0.378	306.900 [265.000,346.000]	283.000 [220.000,335.000]	0.144
SCr (μmol/L)	59.000 [49.100,67.000]	61.000 [49.000,71.000]	0.408	57.800 [48.000,65.300]	59.000 [49.000,68.400]	0.465	60.500 [50.700,68.000]	62.000 [53.000,73.000]	0.413
UACR (mg/g)	0.900 [0.500,2.900]	1.100 [0.500,2.200]	0.880	0.900 [0.600,2.860]	2.000 [0.600,9.500]	0.075	1.360 [0.600,8.780]	2.474 [0.500,8.600]	0.496
eGFR (mL/min/1.73 m^2^)	124.904 [108.435,145.213]	102.838 [86.000,123.980]	<0.001	123.240 [103.339,144.400]	108.811 [89.113,131.861]	0.004	125.161 [106.000,144.444]	100.762 [69.445,121.423]	<0.001
GMI (%)	7.210 [6.390,7.750]	7.490 [6.770,8.050]	0.019	7.160 [6.490,7.845]	7.426 [7.090,8.460]	0.004	7.351 [6.710,7.860]	7.976 [7.270,8.560]	0.024
MBG (mmol/L)	8.076 [7.070,8.700]	8.326 [7.530,9.060]	0.037	8.070 [7.160,8.840]	8.354 [7.910,9.550]	0.007	8.210 [7.460,8.853]	8.860 [7.650,9.410]	0.081
TBR (%)	0.800 [0.300,2.900]	2.100 [1.300,4.260]	<0.001	1.200 [0.300,3.170]	2.200 [1.300,4.300]	<0.001	1.600 [0.300,4.040]	2.000 [1.200,4.700]	0.024
TIR (%)	79.400 [72.400,89.700]	72.400 [60.900,80.400]	<0.001	78.800 [71.400,90.600]	71.200 [59.000,79.500]	<0.001	77.900 [67.790,85.400]	73.100 [60.200,77.200]	0.006
TAR (%)	23.100 [14.400,30.000]	23.700 [15.500,33.600]	0.483	22.900 [13.600,30.220]	23.000 [18.300,35.400]	0.123	24.000 [16.300,31.320]	24.900 [16.280,37.200]	0.457
SD (mmol/L)	2.340 [1.870,2.840]	2.676 [2.200,3.090]	<0.001	2.340 [1.880,2.744]	2.719 [2.260,3.500]	<0.001	2.580 [1.960,2.920]	2.908 [2.270,3.280]	0.079
CV (%)	28.977 [25.413,33.327]	31.250 [27.668,34.995]	0.002	29.007 ± 5.569	32.513 ± 5.073	<0.001	30.398 ± 5.520	32.323 ± 6.036	0.095
MAGE (mmol/L)	5.070 [4.290,6.060]	5.670 [4.980,6.930]	<0.001	5.163 ± 1.401	5.976 ± 1.574	0.001	5.440 [4.468,6.157]	5.620 [4.700,6.130]	0.271
Nc (×10^9^/L)	3.437 [2.860,4.180]	3.540 [3.040,4.450]	0.147	3.610 [3.070,4.178]	3.810 [3.340,4.480]	0.070	3.640 [3.180,4.200]	3.400 [3.000,4.200]	0.940
Lc (×10^9^/L)	1.900 [1.600,2.300]	1.700 [1.500,2.100]	0.005	1.900 [1.600,2.300]	1.700 [1.400,2.230]	0.179	2.000 [1.700,2.300]	1.800 [1.500,2.100]	0.064
PLT (×10^9^/L)	211.000 [188.000,243.000]	223.000 [188.000,247.000]	0.114	209.000 [184.000,233.300]	221.000 [204.000,265.000]	0.012	211.000 [188.000,243.000]	221.000 [191.000,244.000]	0.733
NLR	1.896 [1.504,2.291]	2.082 [1.737,2.580]	<0.001	1.930 [1.607,2.241]	2.088 [1.807,2.742]	0.013	1.900 [1.650,2.206]	2.102 [1.573,2.805]	0.061
SII	382.375 [316.952,505.807]	456.000 [367.173,578.613]	<0.001	389.259 [308.550,479.331]	487.292 [387.279,609.914]	<0.001	373.214 [334.400,467.497]	437.223 [369.307,672.750]	0.018

Data are median [IQR] or mean ± SD. BMI, Body Mass Index; SBP/DBP, Systolic/Diastolic Blood Pressure; FPG, Fasting Plasma Glucose; CP, C-peptide; HbA1c, Glycated Hemoglobin A1c; TC, Total Cholesterol; TG, Triglycerides; HDL-C, High-Density Lipoprotein Cholesterol; LDL-C, Low-Density Lipoprotein Cholesterol; UA, Uric Acid; SCr, Serum Creatinine; UACR, Urine Albumin-to-Creatinine Ratio; eGFR, Estimated Glomerular Filtration Rate; GMI, Glucose Management Indicator; MBG, Mean Blood Glucose; TIR, Time in Range; TBR, Time Below Range; TAR, Time Above Range; SD, Standard Deviation; CV, Coefficient of Variation; NLR, Neutrophil-Lymphocyte Ratio; SII, Systemic Immune-Inflammation Index.

Among initially screened patients, 154 were excluded because essential eligibility or outcome-related information was unavailable, whereas sporadic missingness in retained predictors was handled by multiple imputation. The missingness percentage for each retained predictor is provided in **Supplementary Table 2**. Inter-observer agreement for ultrasound-defined plaque vulnerability was 0.86 (95% CI: 0.81-0.91), indicating excellent agreement before adjudication. Age was analyzed as a continuous variable, and its distribution by plaque group in the training, internal-validation, and external-validation cohorts is presented in **Table 1**.

### Feature selection

**Figure 3:**
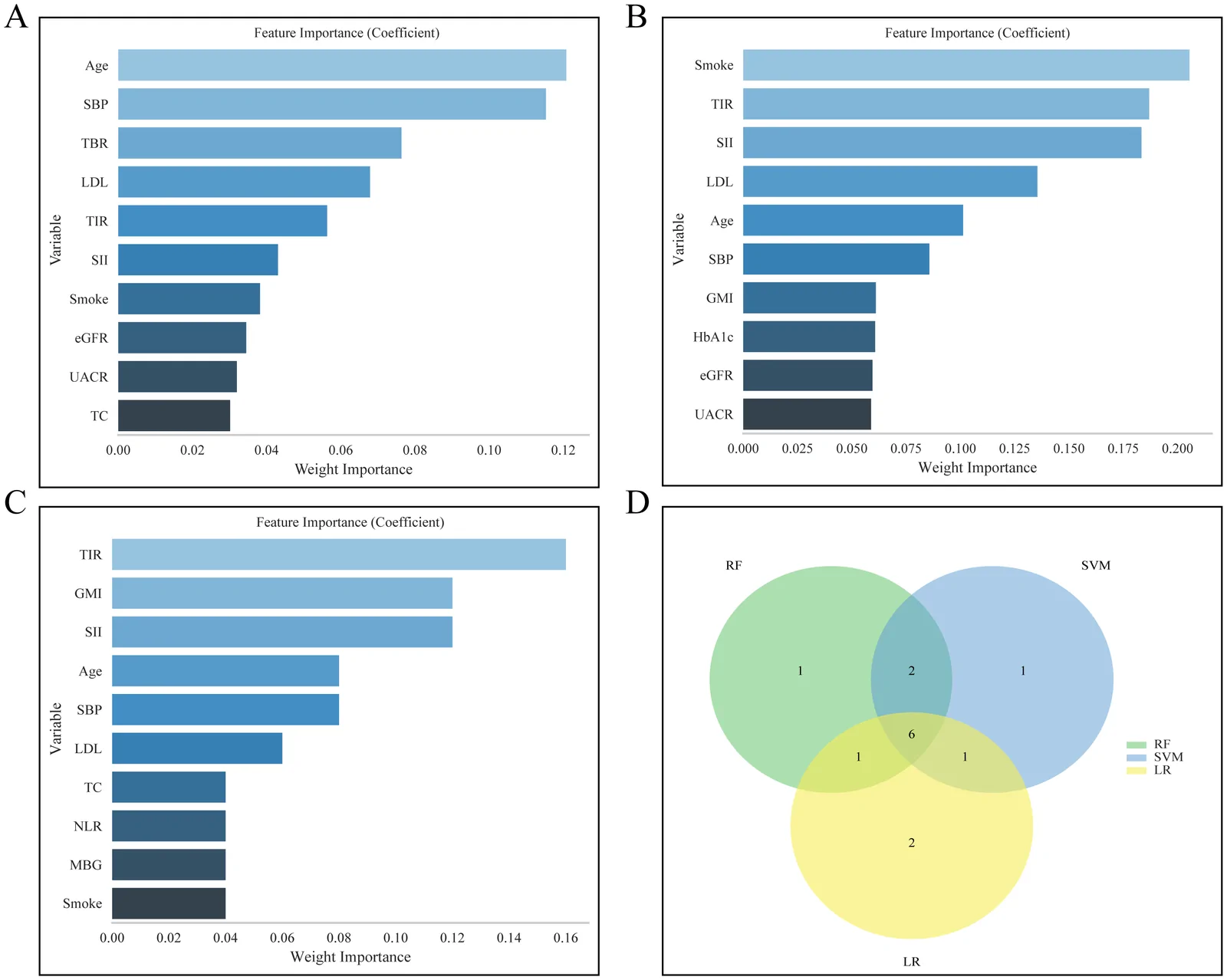
Comparison of feature importance metrics among Logistic Regression, Random Forest, and SVM models. **(A)** Feature importance ranking plot of Logistic Regression; **(B)** Feature importance ranking plot of Random Forest; **(C)** Feature importance ranking plot of SVM; **(D)** Venn diagram showing overlapping core predictors screened by three algorithms.

Following data preprocessing, the importance of features was ranked using multiple algorithms within the training set. The top 10 features identified by each algorithm are shown in **Figures 3A–C**. To validate these findings, a Venn diagram was employed to cross-validate and perform a comprehensive analysis of the screening results across the three algorithms (**Figure 3D**). Consequently, six core feature variables that consistently demonstrated high importance rankings across all three algorithms were chosen as inputs for the prediction models: SBP, LDL-C, age, TIR, SII, and smoking status.

### Model construction and performance evaluation

**Figure 4:**
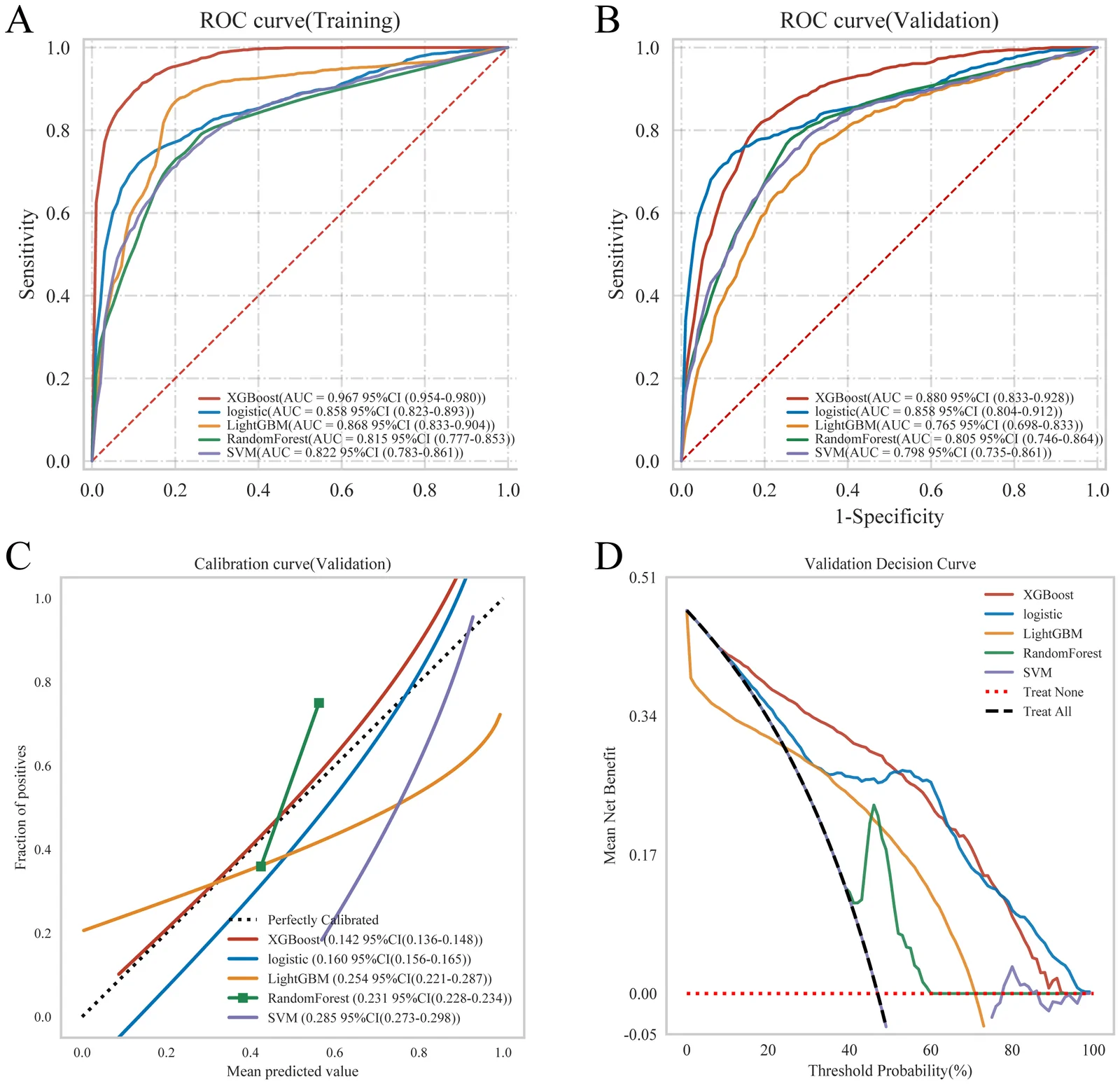
Performance comparison of ML models in the training and internal validation cohorts. **(A, B)** ROC curves. **(C)** Calibration curves and **(D)** DCA.

This investigation comprehensively assessed five ML models through 5-fold cross-validation integrated with grid search. Complete performance metrics are illustrated in **Table 2**, and sensitivity analyses before and after SMOTE are provided in **Supplementary Table 3**. The XGBoost model displayed the best overall performance. In the training dataset, it attained an AUC of 0.967 (95% confidence interval CI: 0.954-0.980), alongside an accuracy of 0.903, sensitivity of 0.896, specificity of 0.909, PPV of 0.898, NPV of 0.909, and F1 score of 0.897. These indicators were higher than those of other models (LR AUC: 0.858; LightGBM AUC: 0.868; RF AUC: 0.815; SVM AUC: 0.822). Internal validation findings showed that the XGBoost model retained good discrimination (AUC: 0.880, 95% CI: 0.833-0.928), although the decline from training to validation performance suggested potential overfitting and supported the need to emphasize validation results rather than training performance alone (**Figure 4**).

**Table 2:** Comparison of model performance metrics.

Model	Dataset	AUC (95% CI)	Accuracy	Sensitivity	Specificity	PPV	NPV	F1 score
XGBoost	Training	0.967 (0.954-0.980)	0.903	0.896	0.909	0.898	0.909	0.897
	Internal validation	0.880 (0.833-0.928)	0.806	0.804	0.875	0.839	0.828	0.793
Logistic	Training	0.858 (0.823-0.893)	0.815	0.724	0.896	0.861	0.786	0.786
	Internal validation	0.858 (0.804-0.912)	0.805	0.727	0.807	0.788	0.785	0.777
LightGBM	Training	0.868 (0.833-0.904)	0.851	0.867	0.838	0.830	0.880	0.846
	Internal validation	0.765 (0.698-0.833)	0.722	0.728	0.716	0.697	0.752	0.709
Random Forest	Training	0.815 (0.777-0.853)	0.775	0.763	0.784	0.759	0.791	0.760
	Internal validation	0.805 (0.746-0.864)	0.755	0.764	0.748	0.729	0.781	0.745
SVM	Training	0.822 (0.783-0.861)	0.771	0.754	0.785	0.757	0.788	0.753
	Internal validation	0.798 (0.735-0.861)	0.732	0.736	0.73	0.707	0.762	0.718

AUC, Area Under the Curve; PPV, Positive Predictive Value; NPV, Negative Predictive Value.

Regarding calibration performance, the calibration curve indicated that the XGBoost model had the highest consistency between predicted probabilities and actual positive event rates among the evaluated models, with its curve closest to the ideal diagonal. This finding was supported by the Brier score: the XGBoost model attained a score of 0.142 (95% CI: 0.136-0.148), which was lower than Logistic Regression (0.160), LightGBM (0.254), Random Forest (0.231), and SVM (0.285), indicating better probability prediction. DCA showed that across a threshold probability range of 20% to 80%, the average net benefit of the XGBoost model remained higher than that of the comparator models.

### External validation

**Figure 5:**
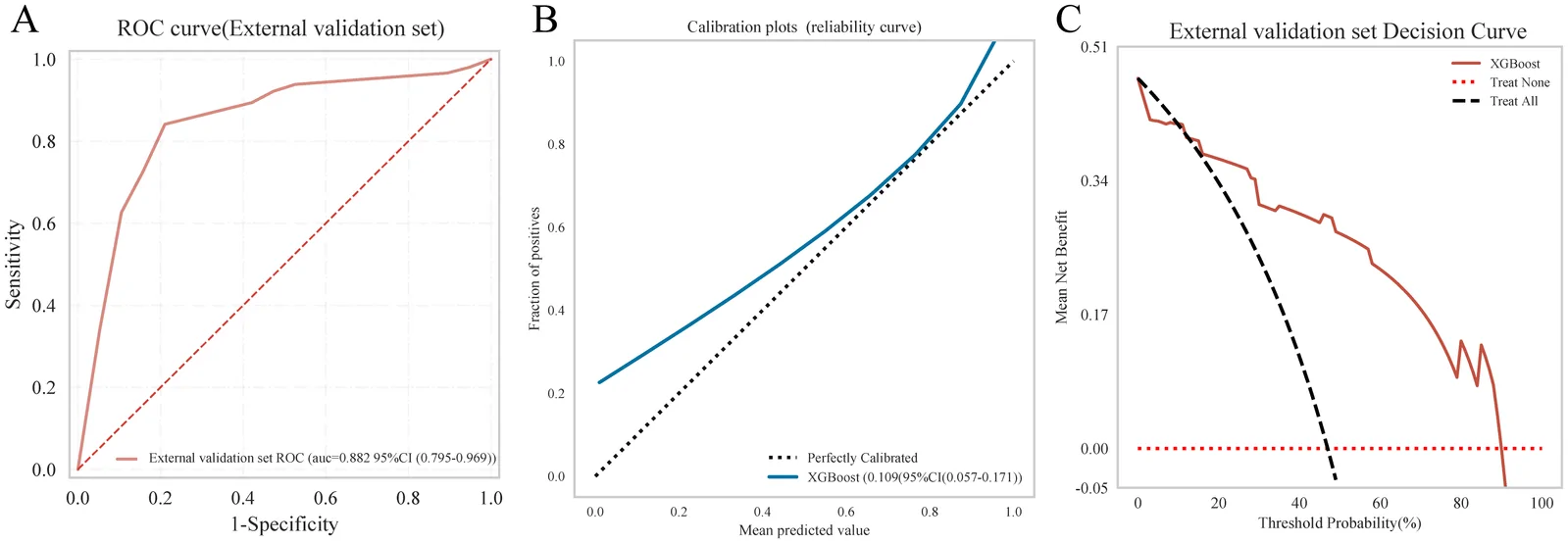
External validation of the XGBoost model. **(A)** ROC curve, **(B)** Calibration plot, and **(C)** DCA for the external validation set.

In the external validation set, the XGBoost model attained an AUC of 0.882 (95% CI: 0.795-0.969), showing good discriminative ability but with a relatively wide confidence interval due to the limited number of vulnerable-plaque cases. The calibration curve remained close to the ideal diagonal, indicating acceptable calibration. DCA revealed that the model’s net benefit was greater than either the ‘treat-all’ or ‘treat-none’ strategy within a threshold range of 0%-80% (**Figure 5**).

### Model interpretability

**Figure 6:**
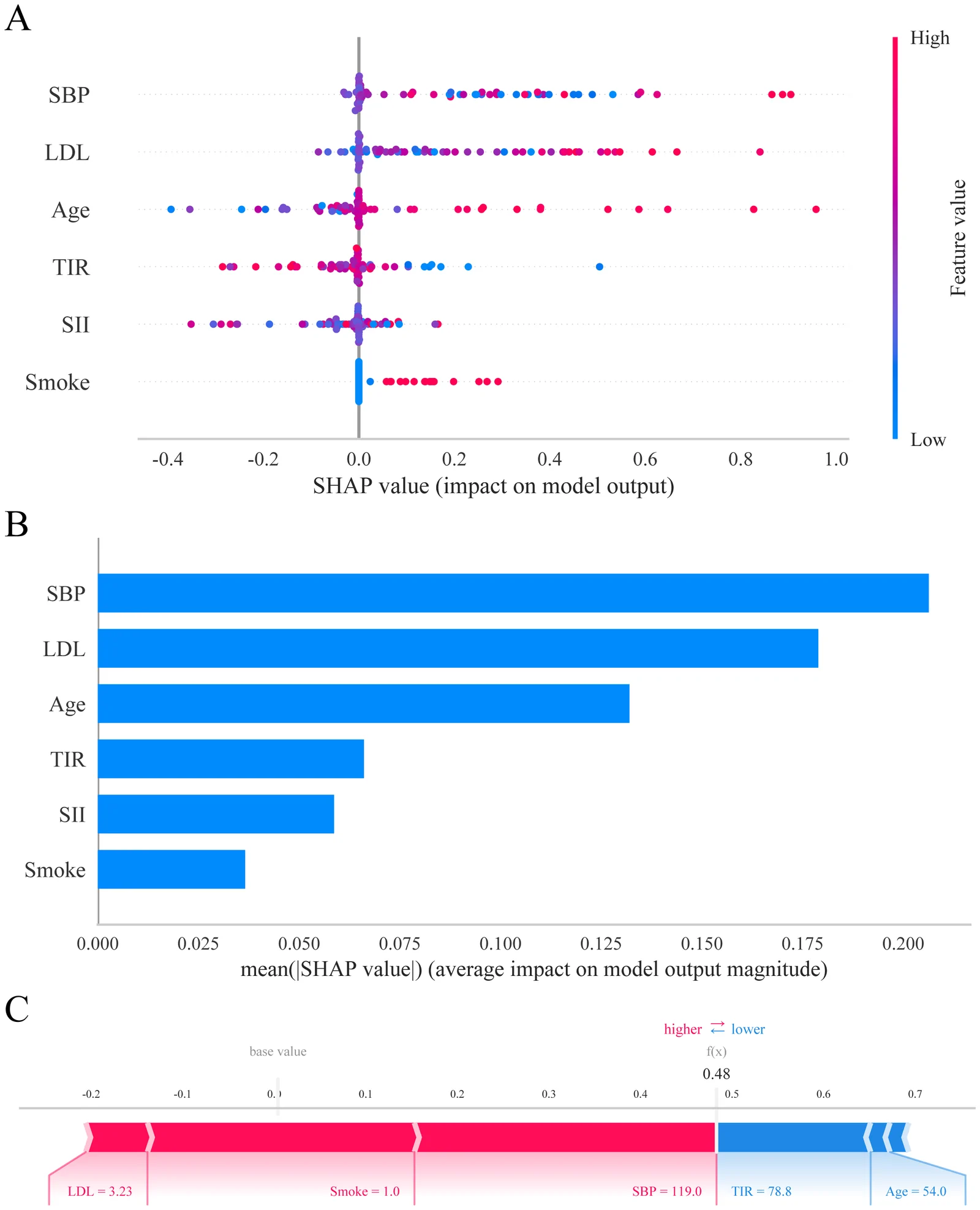
Model interpretation using SHAP. **(A)** SHAP summary plot. **(B)** Mean SHAP values ranking feature importance. **(C)** Force plot illustrating individual prediction logic.

Model interpretability was evaluated using SHAP (SHapley Additive exPlanations), with findings shown in **Figure 6**. **Figure 6A** presents the SHAP feature-contribution beeswarm plot, visually demonstrating how the six features influenced model output in terms of direction and magnitude. Higher SBP, higher LDL-C, older age, higher SII, and smoking were associated with higher predicted probabilities of vulnerable plaques. Conversely, higher TIR values were associated with lower predicted probabilities, suggesting that better CGM-derived glycemic control was associated with plaque stability in this cohort. These SHAP findings should be interpreted as model-based associations rather than causal effects.

**Figure 6B** ranks the features according to mean absolute SHAP values. The predictors, in descending order of importance, were SBP, LDL-C, age, TIR, SII, and smoking status. SBP and LDL-C contributed most strongly to model output. **Figure 6C** presents an individual sample SHAP explanation plot, demonstrating how each feature value contributed to the final prediction probability in a representative case. In this instance, LDL-C level, smoking status, and SBP increased the predicted probability, whereas TIR and younger age decreased the predicted probability. The resulting prediction probability reflected the combined contribution of these features for this individual patient.

## Discussion

Based on a cohort of patients with T2DM and carotid atherosclerotic plaques from two centers, this investigation integrated dynamic glucose parameters, clinical variables, lipid profiles, and inflammatory markers to develop an interpretable XGBoost model for estimating ultrasound-defined plaque vulnerability. Through multi-model cross-screening, six core predictors were identified. The model showed favorable discrimination, calibration, and clinical net benefit in the internal and external validation datasets. Nevertheless, the decline in AUC from the training set to the validation cohorts indicates possible overfitting, and the results should therefore be interpreted primarily according to validation performance. The key clinical rationale is not to replace ultrasound, which remained the reference standard, but to determine whether systemic non-imaging data can enrich risk and help allocate expert plaque-characterization resources. Prospective workflow validation is required before this proposed role can be adopted.

Carotid ultrasound can directly visualize plaque morphology and remains indispensable for confirmation. Nevertheless, diagnostic capability does not eliminate the need for triage when access to experienced vascular sonographers, dedicated plaque characterization, or advanced imaging is constrained. The excellent inter-observer agreement observed in this study reflects two physicians with more than 5 years of cardiovascular-ultrasound experience and should not be assumed to represent all practice settings; prior work has demonstrated meaningful observer variability in duplex plaque characterization (9). Therefore, the proposed value of the model is risk enrichment before expert characterization, not replacement of a readily available definitive test.

We could not identify a nationally representative estimate of carotid-ultrasound screening uptake specifically among Chinese patients with T2DM. Such a rate also cannot be estimated from our cohort because ultrasound examination and confirmed carotid plaque were inclusion prerequisites. We therefore removed any implication that a low screening rate had been demonstrated. Instead, we cite the absence of a universal carotid-ultrasound pathway in current Chinese primary-care diabetes guidance and the lack of support for population-wide screening of asymptomatic adults (10, 11). Whether a model-first strategy reduces cost, waiting time, or unnecessary examinations compared with an ultrasound-first strategy remains unknown and requires prospective health-economic evaluation.

Two distinct use scenarios are now delineated. The primary scenario closest to the present study population is pre-expert-imaging prioritization: among patients with T2DM and established carotid plaque, a higher model-estimated probability could prioritize referral for expert plaque-focused ultrasound or, when clinically appropriate, advanced imaging. A low probability should never cancel clinically indicated imaging. Because TIR is required, the current model is most readily applicable to patients with recent CGM data already available for diabetes-management purposes; the present findings do not support initiating CGM solely to estimate carotid plaque-vulnerability risk. The present study validates discrimination against an expert ultrasound-defined phenotype, but it does not validate the referral workflow or its clinical impact. A secondary post-ultrasound adjunct scenario would use the systemic risk estimate when a basic ultrasound finding is equivocal or local plaque-characterization expertise is limited; this scenario is hypothetical and requires separate prospective validation.

The XGBoost model achieved an AUC of 0.967 in the training set and maintained AUCs of 0.880 and 0.882 in the internal and external validation sets, respectively, suggesting cross-center transportability but also highlighting a training-validation performance gap. Previous ML studies of carotid plaque vulnerability were mainly single-center and did not specifically integrate CGM metrics with systemic inflammatory indices in the high-risk T2DM population (15, 18). A recent nomogram for carotid atherosclerosis in non-obese patients with T2DM incorporated age, sex, SBP, LDL-C, and fasting plasma glucose (26), partly overlapping with our predictors. Other T2DM studies have generally modeled the presence of carotid plaque rather than its vulnerability (27, 28). The present work therefore addresses a more specific phenotype, while its added value over expert imaging remains to be established prospectively.

The significance ranking of the six core features determined via multi-algorithm cross-screening aligns closely with established clinical pathogenic mechanisms. First, SBP and LDL-C emerged as the top two contributors to the model, consistent with current theories of atherogenesis. Hypertension is broadly acknowledged as an independent indicator of atherosclerosis initiation and progression. The underlying mechanism involves abnormal hemodynamic shear stress induced by hypertension, which damages the vascular endothelium, thereby facilitating plaque progression and inflammatory infiltration (29–31). Conversely, LDL-C particles deposit beneath the vascular endothelium to form a lipid core, where they are retained and undergo oxidative modification to become oxidized LDL (oxLDL). Following the massive phagocytosis of oxLDL by macrophages, these cells transform into foam cells, which aggregate and accumulate, ultimately promoting vulnerable atherosclerotic plaque development and advancement (32, 33). Second, as a non-modifiable indicator, age is associated with significant pathological changes in plaque composition. Aging manifests as a reduction in elastic fibers and smooth muscle cells, accompanied by an enlarged lipid core, intraplaque hemorrhage, and increased calcification. These collective changes drive the phenotypic transformation of plaques toward vulnerability (34, 35).

Notably, TIR emerged as an important predictor associated with a lower model-estimated probability of vulnerable plaque. TIR reflects both glycemic exposure and glucose variability (36), information that HbA1c alone cannot fully represent. Reduced TIR may indicate impaired glucose homeostasis, which can intensify oxidative stress and endothelial dysfunction relevant to plaque instability (37, 38). SII integrates peripheral neutrophil, lymphocyte, and platelet counts, thereby summarizing inflammatory, immune-regulatory, and thrombotic components (39). Elevated SII has been associated with vulnerable carotid plaque (40, 41). Smoking, a modifiable risk indicator, is also associated with lipid-rich and vulnerable plaque features (42). Collectively, TIR and SII provide systemic metabolic-inflammatory information that is not contained in morphology alone: ultrasound describes the local plaque phenotype at the time of imaging, whereas the model summarizes systemic conditions associated with that phenotype. This complementarity is pathophysiologically plausible, but whether combined use improves decisions beyond expert ultrasound has not yet been demonstrated.

The substantial age difference between stable- and vulnerable-plaque groups deserves careful interpretation. Age was entered into all models as a continuous variable and ranked third by mean absolute SHAP value, below SBP and LDL-C. This ranking quantifies the contribution of age to model output within the present cohort; it does not indicate causal importance, therapeutic priority, or that a non-modifiable characteristic should outweigh modifiable indicators. SBP, LDL-C, TIR, SII, and smoking remained clinically actionable contributors to the model. Nevertheless, the marked baseline age imbalance may reduce transportability to younger T2DM populations and may reflect residual confounding if age captures cumulative vascular injury, medication exposure, or disease duration. Diabetes duration was not consistently recorded across both centers, so its collinearity with age could not be evaluated. Because the external cohort contained only 29 vulnerable-plaque cases and the age distributions were highly imbalanced, a *post hoc* split at 65 years would produce sparse subgroups and unstable discrimination and calibration estimates. Larger prospective cohorts should prespecify age-stratified validation and assess age jointly with diabetes duration and treatment exposure.

Several clinically recognized variables, including sex and HDL-C, were not selected as final predictors. This does not necessarily indicate that these variables are biologically irrelevant. Rather, in this T2DM cohort, their incremental predictive contribution may have been attenuated after accounting for age, SBP, LDL-C, TIR, SII, and smoking status. Sex-related differences in plaque vulnerability may also be partly mediated by age distribution, hormonal status, smoking exposure, and lipid profiles, while HDL-C concentration alone may not fully reflect HDL function or anti-inflammatory capacity. Homocysteine is a recognized vascular-risk marker and may be related to plaque instability; however, it was not routinely measured across both centers and therefore could not be incorporated without introducing substantial missingness and selection bias. Future studies should include homocysteine, lipoprotein(a), medication exposure, and additional plaque-imaging features to further improve model comprehensiveness.

The “black box” nature of ML represents a barrier to clinical translation. To improve transparency, the present study used SHAP at both global and individual levels to show the direction and relative contribution of each predictor. Individual SHAP plots may support clinician review, identification of discordant risk profiles, and communication of why a model output is high or low. They should not be interpreted as causal explanations or as a basis for individualized treatment without confirmatory imaging and clinical assessment.

Direct numerical comparison with commonly used stroke-risk scores was not undertaken because their target outcomes and eligible populations differ fundamentally from those of the present model. ABCD2 estimates very early stroke risk after transient ischemic attack (43), the Essen Stroke Risk Score estimates recurrent vascular events among patients with previous ischemic stroke or transient ischemic attack (44), and the Framingham Stroke Risk Profile estimates long-term incident stroke risk (45). These tools do not estimate the current presence of an ultrasound-defined vulnerable plaque and generally do not incorporate CGM-derived glucose dynamics or an integrated inflammatory-thrombotic index such as SII. The present model instead addresses a T2DM-specific, cross-sectional plaque-vulnerability phenotype and combines traditional vascular risk factors with systemic metabolic-inflammatory information. Several variables required by the traditional scores were also unavailable. Consequently, NRI, IDI, calibration comparison, and comparative DCA would not be interpretable across these different outcomes and time horizons. Logistic Regression using the same predictors and endpoint was retained as the conventional statistical benchmark. The present results do not establish incremental value over validated stroke-prognosis scores, a future plaque-vulnerability-specific score, or expert ultrasound.

At the current evidence stage, no universal clinical action threshold is justified. We therefore provide a prospective threshold-selection framework rather than a *post hoc* numerical cutoff. A sensitivity-prioritized operating point, a balanced operating point based on the maximum Youden index, and a high-specificity escalation flag may each be evaluated according to the intended workflow. In this framework, sensitivity ≥90% is only a hypothetical planning example corresponding to a miss rate of no more than 10%; it was not derived from a formal harm-minimization analysis, screening-program precedent, or stakeholder consensus and should not be interpreted as a clinically endorsed universal target. An implementation study may reasonably select a lower or higher sensitivity target after explicitly weighing the harm of missed vulnerable plaques against false-positive referrals and considering local prevalence, stroke burden, ultrasound capacity, waiting times, and patient and clinician preferences. Similarly, a specificity target of ≥95% may be explored for an expedited expert-imaging flag, but not for direct treatment. Each candidate threshold should be reported with sensitivity, specificity, PPV, NPV, calibration, the number referred per 100 patients, the number of vulnerable plaques missed, and decision-curve net benefit. Because the external cohort contained only 29 vulnerable-plaque cases and no intervention pathway was prespecified, numerical cutoffs derived *post hoc* from the current data would be unstable and potentially optimistic; they require prospective validation.

False-positive triage referrals are not harmless. Being labeled as potentially harboring a vulnerable plaque may cause anxiety, and incidental or equivocal findings may lead to overdiagnosis or a diagnostic cascade involving repeat ultrasound, neurology consultation, CTA, MRI, or other testing. These downstream procedures can add cost and, where applicable, radiation or contrast-related risk. The expert plaque-focused ultrasound proposed here uses conventional B-mode and color-Doppler techniques and does not itself require microbubble contrast; CEUS would be a separate advanced examination. False-positive referrals also consume specialist imaging slots and may delay assessment of patients with stronger clinical indications, an important opportunity cost in capacity-constrained settings.

The clinical and economic feasibility of the model will vary across settings. It depends on local CGM uptake, whether recent CGM data are already available, the marginal cost of obtaining and processing CGM data, and the availability, examination time, specialist expertise, and cost of the expert plaque-focused ultrasound being prioritized. Secondary use of CGM data already collected for diabetes management may entail little or no additional acquisition cost, whereas initiating CGM solely for this model would materially change its resource requirements and is not supported by the present findings. Other barriers include the short 72-hour monitoring window, inter-institutional differences in laboratory assays and units, missing or incorrectly mapped EHR variables, and the need for local calibration. The current six-predictor model is therefore most feasible where recent CGM data are already available. A reduced model without TIR should be separately developed and externally validated for settings with low CGM uptake. Deployment would require automated data-quality checks, version control, calibration-drift monitoring, periodic updating, and clinical governance. No formal health-economic evaluation was performed. A future implementation study should compare model-guided prioritization with usual care or an ultrasound-first strategy, measuring referrals, waiting time, false-positive referrals and their downstream consequences, vulnerable plaques missed, cost per correctly identified case, and downstream clinical outcomes. A public calculator was not released because the current retrospective model has not undergone prospective impact evaluation and lacks a validated clinical threshold. Any future research-use interface should use a locked and versioned model, clearly display its non-diagnostic status and uncertainty, avoid collection of direct identifiers, minimize data retention, and comply with applicable privacy and cybersecurity requirements.

## Limitations

First, this investigation adopted a retrospective cross-sectional design; therefore, the model identifies ultrasound-defined plaque vulnerability but does not establish causality or predict future stroke events. Second, all participants had undergone carotid ultrasound and had confirmed plaque, so the model has not been validated for universal upstream screening of previously unscreened patients with T2DM. The study also cannot estimate carotid-ultrasound uptake in the broader Chinese T2DM population. Third, plaque determination was based on conventional carotid ultrasound rather than pathology, CTA, MRI (46, 47), or CEUS. Although all images were independently assessed by two experienced physicians and inter-observer agreement was quantified, ultrasound remains operator-dependent and has limited ability to characterize intraplaque hemorrhage, fibrous-cap rupture, lipid-rich necrotic cores, and neovascularization. Fourth, external validation was derived from a single center and included only 29 vulnerable-plaque cases, limiting the precision of AUC, calibration, DCA, subgroup estimates, and threshold-specific performance. The study was therefore underpowered for reliable age-stratified validation or selection of a clinical cutoff. Fifth, CGM monitoring was limited to 72 hours, which may not fully capture long-term glycemic variability. Although all CGM records in this study had been obtained for routine clinical diabetes-management assessment, the current model may be less feasible where recent CGM data are unavailable; these findings do not justify initiating CGM solely for plaque-risk prediction. Sixth, some potentially relevant factors, including homocysteine, diabetes duration, antiplatelet or statin therapy, and detailed plaque-imaging biomarkers, were unavailable or incompletely recorded; collinearity between age and diabetes duration could not be assessed. Seventh, established scores such as ABCD2, the Essen Stroke Risk Score, and the Framingham Stroke Risk Profile target different outcomes and time horizons, and no validated plaque-vulnerability-specific score was available for a same-outcome comparison. Finally, no formal health-economic evaluation, model-first referral pathway, clinical action threshold, impact study, or public calculator has been validated. Despite cross-validation and external validation, the training-validation performance gap suggests possible overfitting. Prospective multicenter workflow and outcome validation, local recalibration, context-specific threshold evaluation, development and validation of a reduced model without TIR, and formal health-economic analysis are required before deployment.

## Conclusion

In conclusion, this investigation developed and externally validated an interpretable XGBoost model based on six clinical, laboratory, and CGM-derived indicators for estimating ultrasound-defined carotid plaque vulnerability among patients with T2DM and established carotid plaque. Its clinical rationale is not to replace ultrasound, but to provide a systemic non-imaging risk estimate that may help prioritize expert plaque characterization when capacity or expertise is constrained, particularly when recent CGM data are already available for diabetes management. The model should not currently be used to justify initiating CGM solely for plaque-risk prediction, exclude clinically indicated imaging, determine treatment, trigger specialist intervention, or set surveillance intervals. Prospective multicenter validation should evaluate the proposed workflow, locally justified sensitivity- and specificity-based thresholds, incremental value alongside ultrasound, calibration monitoring, patient outcomes, a reduced model without TIR, and cost-effectiveness.

## Data Availability

The original contributions presented in the study are included in the article/**Supplementary Material**. Further inquiries can be directed to the corresponding author.
