# Supplementary material for: Prediction of carotid vulnerable plaques in patients with type 2 diabetes using interpretable machine learning models
Source: Front Endocrinol (Lausanne). 2026 Aug 18;17:1911126. doi: 10.3389/fendo.2026.1911126 (PMC13529566; doi:10.3389/fendo.2026.1911126)
Supplement: Supplementary file 1 [file Table1.docx]

| Library/Package | Version | Primary Functions Used | Official Website |
| --- | --- | --- | --- |
| Python | 3.9+ | Base programming language environment | https://www.python.org/ |
| NumPy | 1.23 | Basic numerical computation and array manipulation | https://numpy.org/ |
| Pandas | 1.5.3 | Data cleaning, multiple imputation, and missing data handling | https://pandas.pydata.org/ |
| Scikit-learn | 1.3.0 | Feature standardization, Logistic Regression, SVM, Random Forest, grid search, and 5-fold cross-validation | https://scikit-learn.org/ |
| Imbalanced-learn | 0.10.1 | Handling class imbalance using the SMOTE algorithm | https://imbalanced-learn.org/ |
| XGBoost | 1.7.6 | Building XGBoost gradient boosting tree models | https://xgboost.readthedocs.io/ |
| LightGBM | 4.0.0 | Building LightGBM efficient gradient boosting models | https://lightgbm.readthedocs.io/ |
| SHAP | 0.41.0 | Calculating SHAP values for global and local interpretability analysis | https://shap.readthedocs.io/ |
| Matplotlib | 3.7.2 | Plotting calibration curves and basic visualizations | https://matplotlib.org/ |
| Decision Curve | latest | Performing Decision Curve Analysis (DCA) to evaluate net benefit | https://github.com/yongzhuang/Decision-curve-analysis |

Table S1: Summary of software libraries, versions, and purposes used in this study

Table S2. Missingness summary for candidate predictors and handling strategy.

| **Variable** | **Overall missingness, n (%)** | **Training/internal cohort missingness, n (%)** | **External cohort missingness, n (%)** | **Handling** |
| --- | --- | --- | --- | --- |
| Age | 0 (0.0) | 0 (0.0) | 0 (0.0) | Retained and imputed (missingness <=20%) |
| Sex | 0 (0.0) | 0 (0.0) | 0 (0.0) | Retained and imputed (missingness <=20%) |
| BMI | 7 (0.8) | 5 (0.7) | 2 (1.1) | Retained and imputed (missingness <=20%) |
| Smoking history | 0 (0.0) | 0 (0.0) | 0 (0.0) | Retained and imputed (missingness <=20%) |
| Drinking history | 3 (0.3) | 2 (0.3) | 1 (0.6) | Retained and imputed (missingness <=20%) |
| SBP | 0 (0.0) | 0 (0.0) | 0 (0.0) | Retained and imputed (missingness <=20%) |
| DBP | 0 (0.0) | 0 (0.0) | 0 (0.0) | Retained and imputed (missingness <=20%) |
| FPG | 6 (0.7) | 5 (0.7) | 1 (0.6) | Retained and imputed (missingness <=20%) |
| CP | 22 (2.5) | 18 (2.5) | 4 (2.3) | Retained and imputed (missingness <=20%) |
| HbA1c | 5 (0.6) | 4 (0.6) | 1 (0.6) | Retained and imputed (missingness <=20%) |
| TC | 4 (0.5) | 3 (0.4) | 1 (0.6) | Retained and imputed (missingness <=20%) |
| TG | 4 (0.5) | 3 (0.4) | 1 (0.6) | Retained and imputed (missingness <=20%) |
| HDL-C | 4 (0.5) | 3 (0.4) | 1 (0.6) | Retained and imputed (missingness <=20%) |
| LDL-C | 4 (0.5) | 3 (0.4) | 1 (0.6) | Retained and imputed (missingness <=20%) |
| UA | 11 (1.2) | 8 (1.1) | 3 (1.7) | Retained and imputed (missingness <=20%) |
| SCr | 3 (0.3) | 2 (0.3) | 1 (0.6) | Retained and imputed (missingness <=20%) |
| eGFR | 3 (0.3) | 2 (0.3) | 1 (0.6) | Retained and imputed (missingness <=20%) |
| UACR | 38 (4.3) | 31 (4.4) | 7 (4.0) | Retained and imputed (missingness <=20%) |
| GMI | 0 (0.0) | 0 (0.0) | 0 (0.0) | Retained and imputed (missingness <=20%) |
| TIR | 0 (0.0) | 0 (0.0) | 0 (0.0) | Retained and imputed (missingness <=20%) |
| TAR | 0 (0.0) | 0 (0.0) | 0 (0.0) | Retained and imputed (missingness <=20%) |
| TBR | 0 (0.0) | 0 (0.0) | 0 (0.0) | Retained and imputed (missingness <=20%) |
| MBG | 0 (0.0) | 0 (0.0) | 0 (0.0) | Retained and imputed (missingness <=20%) |
| SD | 0 (0.0) | 0 (0.0) | 0 (0.0) | Retained and imputed (missingness <=20%) |
| CV | 0 (0.0) | 0 (0.0) | 0 (0.0) | Retained and imputed (missingness <=20%) |
| MAGE | 0 (0.0) | 0 (0.0) | 0 (0.0) | Retained and imputed (missingness <=20%) |
| Lc | 5 (0.6) | 4 (0.6) | 1 (0.6) | Retained and imputed (missingness <=20%) |
| Nc | 5 (0.6) | 4 (0.6) | 1 (0.6) | Retained and imputed (missingness <=20%) |
| PLT | 5 (0.6) | 4 (0.6) | 1 (0.6) | Retained and imputed (missingness <=20%) |
| NLR | 5 (0.6) | 4 (0.6) | 1 (0.6) | Retained and imputed (missingness <=20%) |
| SII | 5 (0.6) | 4 (0.6) | 1 (0.6) | Retained and imputed (missingness <=20%) |
| Homocysteine | 545 (61.7) | 431 (61.0) | 114 (64.4) | Excluded from modeling because missingness >20% and not routinely measured in both centers |

Table S3. Sensitivity analysis comparing model performance before and after SMOTE.

| Model | Dataset | SMOTE in training | AUC (95% CI) | Accuracy | Sensitivity | Specificity | PPV | NPV | F1-score |
| --- | --- | --- | --- | --- | --- | --- | --- | --- | --- |
| XGBoost | Training | No | 0.943 (0.925-0.961) | 0.871 | 0.714 | 0.906 | 0.663 | 0.933 | 0.688 |
| XGBoost | Training | Yes | 0.967 (0.954-0.980) | 0.903 | 0.896 | 0.909 | 0.898 | 0.909 | 0.897 |
| XGBoost | Internal validation | No | 0.858 (0.805-0.911) | 0.812 | 0.628 | 0.859 | 0.529 | 0.902 | 0.574 |
| XGBoost | Internal validation | Yes | 0.880 (0.833-0.928) | 0.806 | 0.804 | 0.875 | 0.839 | 0.828 | 0.793 |
| XGBoost | External validation | No | 0.861 (0.773-0.949) | 0.802 | 0.621 | 0.838 | 0.429 | 0.919 | 0.507 |
| XGBoost | External validation | Yes | 0.882 (0.795-0.969) | 0.797 | 0.759 | 0.804 | 0.431 | 0.944 | 0.550 |

Table S4. Bootstrap confidence intervals and optimism-corrected estimates for key model-performance metrics.

| Model | Dataset | Metric | Apparent estimate | Bootstrap mean | Optimism-corrected estimate | 95% CI |
| --- | --- | --- | --- | --- | --- | --- |
| XGBoost | Training | AUC | 0.967 | 0.960 | 0.960 | 0.945-0.975 |
| XGBoost | Training | Accuracy | 0.903 | 0.894 | 0.895 | 0.867-0.921 |
| XGBoost | Training | Sensitivity | 0.896 | 0.873 | 0.873 | 0.813-0.927 |
| XGBoost | Training | Specificity | 0.909 | 0.902 | 0.902 | 0.872-0.931 |
| XGBoost | Training | Brier score | 0.118 | 0.126 | 0.126 | 0.105-0.149 |
| XGBoost | Internal validation | AUC | 0.880 | 0.872 | 0.872 | 0.833-0.928 |
| XGBoost | Internal validation | Accuracy | 0.806 | 0.798 | 0.798 | 0.744-0.852 |
| XGBoost | Internal validation | Sensitivity | 0.804 | 0.781 | 0.781 | 0.651-0.884 |
| XGBoost | Internal validation | Specificity | 0.875 | 0.861 | 0.861 | 0.801-0.915 |
| XGBoost | Internal validation | Brier score | 0.142 | 0.146 | 0.146 | 0.136-0.148 |
| XGBoost | External validation | AUC | 0.882 | 0.875 | 0.875 | 0.795-0.969 |
| XGBoost | External validation | Accuracy | 0.797 | 0.789 | 0.789 | 0.729-0.853 |
| XGBoost | External validation | Sensitivity | 0.759 | 0.738 | 0.738 | 0.586-0.879 |
| XGBoost | External validation | Specificity | 0.804 | 0.795 | 0.795 | 0.730-0.859 |
| XGBoost | External validation | Brier score | 0.109 | 0.113 | 0.113 | 0.057-0.171 |
